# Supplementary material for: Multiplexed proteomics of autophagy-deficient murine macrophages reveals enhanced antimicrobial immunity via the oxidative stress response
Source: eLife. 2021 Jun 4;10:e62320. doi: 10.7554/eLife.62320 (PMC8177894; doi:10.7554/eLife.62320)
Supplement: Supplementary file 2. [file elife-62320-supp2.docx]

| **Supplementary File 2** | | |
| --- | --- | --- |
| **Protein name/site** | | **Biological significance** |
| **ATG16L1** | |  |
| S287, S289 | | - Phosphorylation at these sites is regulated by ULK1 and IKK kinases(Alsaadi et al., 2019; Diamanti et al., 2017). Important for ATG16L1 localization to Salmonella |
| **ATG5** | |  |
| K234 | | - Novel, no reference found |
| **ATG12** | |  |
| No sites reported in our study. | |  |
| **ATG3** | |  |
| K71 | | - N/A, not novel |
| **ATG7** | |  |
| K419 | | - N/A, not novel |
| **ATG4A** | |  |
| No sites reported in our study. | |  |
| **ATG4B** | |  |
| K154 | | - N/A, not novel |
| **MLP3A/LC3** | |  |
| K39 | | - Novel, no reference found |
| K42 | | - N/A, not novel |
| **TAX1BP1** | |  |
| S225 | | - N/A, not novel |
| T426 | | - Novel, no reference found |
| T494 | | - Novel, no reference found |
| S632 | | - N/A, not novel |
| S637 | | - N/A, not novel |
| S693 | | - Shembade et al.(Shembade et al., 2011) described that IKKβ phosphorylates this site, which regulates Tax1BP1, RNF11, A20 assembly. This complex mediates negative feedback of NF-kB signaling |
| S708 | | - N/A, not novel |
| K284 | | - Novel, no reference found |
| K311 | | - Novel, no reference found |
| K479 | | - N/A, not novel |
| K536 | | - Novel, no reference found |
| K551 | | - Novel, no reference found |
| K563 | | - N/A, not novel |
| K618 | | - N/A, not novel |
| K624 | | - Novel, no reference found |
| K627 | | - N/A, not novel |
| **p62/SQSTM1** | |  |
| S24 | | - N/A, not novel |
| S28 | | - Xu et al.(Xu et al., 2019) reported p62 phosphorylation at S28 by fructokinase A in response to oxidative stress. This PTM subsequently blocks p62 ubiquitylation, resulting in p62 aggregation with Keap1 and activation of Nrf2 |
| S207 | | - N/A, not novel |
| T221 | | - N/A, not novel |
| S224 | | - N/A, not novel |
| S226 | | - N/A, not novel |
| S266 | | - N/A, not novel |
| T269 and T272 | | - Linares et al.(Linares et al., 2015) showed that p38/MEKK3 mediates phosphorylation at these sites. This results in TRAF6 recruitment and promotes mTORC1 translocation to lysosomes - Linares et al.(Linares et al., 2011) showed that T269 and S272 are CDK1-regulated sites. Important for cell proliferation |
| S271 | | - N/A, not novel |
| S276 | | - N/A, not novel |
| S277 | | - N/A, not novel |
| S278 | | - N/A, not novel |
| T280 | | - Novel, no reference found |
| S284 | | - N/A, not novel |
| T286, S290, S367, S368 | | - Hu et al.(Hu et al., 2020) reported that phosphorylation at these sites requires TBK1 |
| S289 | | - N/A, not novel |
| S292 | | - Novel, no reference found |
| S293 | | - N/A, not novel |
| S308 | | - Novel, no reference found |
| S334 | | - N/A, not novel |
| K13 | | - Song et al.(Song et al., 2016) showed that K13 is ubiquitylated by Parkin, leading to p62 degradation |
| K157 | | - N/A, not novel |
| K422 | | - Lee et al.(Lee et al., 2017) showed ubiquitylation at this site by KEAP1, which is required for p62 receptor function |
| K437 | | - N/A, not novel |
| **NBR1** | |  |
| T6 | | - Novel, no reference found |
| S657 | | - N/A, not novel |
| K538 | | - N/A, not novel |
| **CACO1** | |  |
| S563 | | - N/A, not novel |
| **OPTN** | |  |
| S184 | | - N/A, not novel |
| S187 | | - Phosphorylation at this site requires TBK1 in response to LPS in macrophages. These events enhance OPTN binding to ubiquitin chains and LC3, which is important for mitophagy and xenophagy (Gleason et al., 2011; Heo et al., 2015; Lazarou et al., 2015; Moore and Holzbaur, 2016; Richter et al., 2016; Wild et al., 2011) |
| T210 | | - N/A, not novel |
| S218 | | - N/A, not novel |
| S530 | | - N/A, not novel |
| S547 | | - N/A, not novel |
| S556 | | - N/A, not novel |
| **TOLIP** | |  |
| K63 | | - N/A, not novel |
| K66 | | - N/A, not novel |
| K143 | | - N/A, not novel |
| **RETR3/FAM143c** | |  |
| S26 | | - N/A, not novel |
| S28 | | - N/A, not novel |
| T307 | | - N/A, not novel |
| T310 | | - N/A, not novel |
| S313 | | - N/A, not novel |
| S320 | | - N/A, not novel |
| S360 | | - N/A, not novel |
| **FUND2** | |  |
| K23 | | - N/A, not novel |
| K30 | | - N/A, not novel |
| K114 | | - Novel, no reference found |
| K121 | | - Novel, no reference found |
| K123 | | - N/A, not novel |
| **TBC17** | |  |
| T19 | | - N/A, not novel |
| S20 | | - N/A, not novel |
| S176 | | - Novel, no reference found |
| K23 | | - N/A, not novel |
| K105 | | - Novel, no reference found |
| **MTOR** | | |
| S2454 | - Tao et al. (Tao et al., 2010) reported autophosphorylation at this site that is important for mTOR function | |
| K1218 | - N/A, not novel | |
| **ULK2** |  | |
| S430 | - N/A, not novel | |
| T531 | - N/A, not novel | |
| S600 | - N/A, not novel | |
| **ATGA1/ATG101** | | |
| No sites reported |  | |
| **RBCC1/FIP200** | | |
| S222 | - N/A, not novel | |
| S237 | - N/A, not novel | |
| T238 | - N/A, not novel | |
| S243 | - N/A, not novel | |
| T252 | - N/A, not novel | |
| S253 | - N/A, not novel | |
| S257 | - N/A, not novel | |
| S261 | - N/A, not novel | |
| T642 | - Novel, no reference found | |
| S646 | - N/A, not novel | |
| S649 | - N/A, not novel | |
| S652 | - N/A, not novel | |
| S666 | - N/A, not novel | |
| **BECN1** | | |
| No sites reported |  | |
| **BAKOR/ATG14** | | |
| No sites reported |  | |
| **PK3C3/VPS34** | | |
| S164 | - Phosphorylation at S164 occurs in myoblasts treated with EtOH and correlates with increased autophagy flux(Hong-Brown et al., 2017) - Wen et al.(Wen et al., 2015) reported an increase of S164 phosphorylation upon treatment of ovarian cancer spheroids with a monoclonal antibody against folate receptor alpha | |
| S165 | - Kim et al.(Kim et al., 2013) identified phosphorylation at this residue by AMP. This leads to inhibition of the non-autophagy Vps34 complex and suppression of overall PI(3)P production, which correlates with induction of autophagy | |
| **PI3R4/VPS15** | | |
| S813 | - N/A, not novel | |
| S853 | - N/A, not novel | |
| S892 | - N/A, not novel | |
| S894 | - N/A, not novel | |
| S895 | - N/A, not novel | |
| S903 | - Novel, no reference found | |
| T904 | - Novel, no reference found | |
| S905 | - N/A, not novel | |
| K1309 | - N/A, not novel | |
| **WIPI2** | | |
| S394 | - N/A, not novel | |
| S395 | - N/A, not novel | |
| **WIPI4** | | |
| No sites reported |  | |
| **UVRAG** | | |
| S481 | - N/A, not novel | |
| S482 | - N/A, not novel | |
| T483 | - N/A, not novel | |
| S492 | - mTORC1-regulated site(Kim et al., 2015) | |
| S497 | - mTORC1-regulated site(Kim et al., 2015). This enhances UVRAG interaction with RUBICON, resulting in inhibition of autophagosome maturation | |
| T517 | - mTORC1-regulated site(Kim et al., 2015) | |
| S521 | - Phosphorylation at this site by CSNK1A1 disrupts SMURF1 binding to UVRAG and blocks UVRAG ubiquitylation that is needed to promote autophagosome maturation(Feng et al., 2019) | |
| S548 | - mTORC1-regulated site(Kim et al., 2015) | |
| S549 | - mTORC1-regulated site(Kim et al., 2015) | |
| S688 | - N/A, not novel | |
| **RUBIC** | | |
| S198 | - N/A, not novel | |
| S250 | - N/A, not novel | |
| S252 | - Novel, no reference found | |
| S370 | - N/A, not novel | |
| S380 | - N/A, not novel | |
| S389 | - N/A, not novel | |
| S390 | - N/A, not novel | |
| S392 | - N/A, not novel | |
| S412 | - N/A, not novel | |
| S552 | - Novel, no reference found | |
| S554 | - Novel, no reference found | |
| S558 | - N/A, not novel | |
| **ATG9A** | | |
| S18 | - N/A, not novel | |
| **ATG2B** | | |
| S239 | - N/A, not novel | |
| S240 | - N/A, not novel | |
| S255 | - N/A, not novel | |
| S379 | - N/A, not novel | |
| T398 | - N/A, not novel | |
| S401 | - Novel, no reference found | |
| S484 | - N/A, not novel | |
| S494 | - N/A, not novel | |
| S885 | - N/A, not novel | |
| S1395 | - N/A, not novel | |
| T1570 | - Novel, no reference found | |
| S1571 | - N/A, not novel | |
| S1741 | - N/A, not novel | |

**References**

Alsaadi, R.M., Losier, T.T., Tian, W., Jackson, A., Guo, Z., Rubinsztein, D.C., and Russell, R.C. (2019). ULK1-mediated phosphorylation of ATG16L1 promotes xenophagy, but destabilizes the ATG16L1 Crohn’s mutant. EMBO Rep. *20*, e46885.

Diamanti, M.A., Gupta, J., Bennecke, M., De Oliveira, T., Ramakrishnan, M., Braczynski, A.K., Richter, B., Beli, P., Hu, Y., Saleh, M., et al. (2017). IKKα controls ATG16L1 degradation to prevent ER stress during inflammation. J. Exp. Med. *214*, 423–437.

Feng, X., Jia, Y., Zhang, Y., Ma, F., Zhu, Y., Hong, X., Zhou, Q., He, R., Zhang, H., Jin, J., et al. (2019). Ubiquitination of UVRAG by SMURF1 promotes autophagosome maturation and inhibits hepatocellular carcinoma growth. Autophagy *15*, 1130–1149.

Gleason, C.E., Ordureau, A., Gourlay, R., Arthur, J.S.C., and Cohen, P. (2011). Polyubiquitin binding to optineurin is required for optimal activation of TANK-binding kinase 1 and the production of interferon β. J. Biol. Chem. jbc.M111.267567.

Heo, J.-M., Ordureau, A., Paulo, J.A., Rinehart, J., and Harper, J.W. (2015). The PINK1-PARKIN Mitochondrial Ubiquitylation Pathway Drives a Program of OPTN/NDP52 Recruitment and TBK1 Activation to Promote Mitophagy. Mol. Cell *60*, 7–20.

Hong-Brown, L.Q., Brown, C.R., Navaratnarajah, M., and Lang, C.H. (2017). FOXO1-AMPK-ULK1 Regulates Ethanol-induced Autophagy in Muscle by Enhanced ATG14 Association with the BECN1-PIK3C3 Complex. Alcohol. Clin. Exp. Res. *41*, 895–910.

Hu, L., Xie, H., Liu, X., Potjewyd, F., James, L.I., Wilkerson, E.M., Herring, L.E., Xie, L., Chen, X., Cabrera, J.C., et al. (2020). TBK1 Is a Synthetic Lethal Target in Cancer with VHL Loss. Cancer Discov. *10*, 460–475.

Kim, J., Kim, Y.C., Fang, C., Russell, R.C., Kim, J.H., Fan, W., Liu, R., Zhong, Q., and Guan, K.-L. (2013). Differential Regulation of Distinct Vps34 Complexes by AMPK in Nutrient Stress and Autophagy. Cell *152*, 290–303.

Kim, Y.-M., Jung, C.H., Seo, M., Kim, E.K., Park, J.-M., Bae, S.S., and Kim, D.-H. (2015). mTORC1 Phosphorylates UVRAG to Negatively Regulate Autophagosome and Endosome Maturation. Mol. Cell *57*, 207–218.

Lazarou, M., Sliter, D.A., Kane, L.A., Sarraf, S.A., Wang, C., Burman, J.L., Sideris, D.P., Fogel, A.I., and Youle, R.J. (2015). The ubiquitin kinase PINK1 recruits autophagy receptors to induce mitophagy. Nature *524*, 309–314.

Lee, Y., Chou, T.-F., Pittman, S.K., Keith, A.L., Razani, B., and Weihl, C.C. (2017). Keap1/Cullin3 Modulates p62/SQSTM1 Activity via UBA Domain Ubiquitination. Cell Rep. *19*, 188–202.

Linares, J.F., Amanchy, R., Greis, K., Diaz-Meco, M.T., and Moscat, J. (2011). Phosphorylation of p62 by cdk1 Controls the Timely Transit of Cells through Mitosis and Tumor Cell Proliferation. Mol. Cell. Biol. *31*, 2171–2171.

Linares, J.F., Duran, A., Reina-Campos, M., Aza-Blanc, P., Campos, A., Moscat, J., and Diaz-Meco, M.T. (2015). Amino Acid Activation of mTORC1 by a PB1-Domain-Driven Kinase Complex Cascade. Cell Rep. *12*, 1339–1352.

Moore, A.S., and Holzbaur, E.L.F. (2016). Dynamic recruitment and activation of ALS-associated TBK1 with its target optineurin are required for efficient mitophagy. Proc. Natl. Acad. Sci. U. S. A. *113*, E3349-3358.

Richter, B., Sliter, D.A., Herhaus, L., Stolz, A., Wang, C., Beli, P., Zaffagnini, G., Wild, P., Martens, S., Wagner, S.A., et al. (2016). Phosphorylation of OPTN by TBK1 enhances its binding to Ub chains and promotes selective autophagy of damaged mitochondria. Proc. Natl. Acad. Sci. *113*, 4039–4044.

Shembade, N., Pujari, R., Harhaj, N.S., Abbott, D.W., and Harhaj, E.W. (2011). The kinase IKKα inhibits activation of the transcription factor NF-κB by phosphorylating the regulatory molecule TAX1BP1. Nat. Immunol. *12*, 834–843.

Song, P., Li, S., Wu, H., Gao, R., Rao, G., Wang, D., Chen, Z., Ma, B., Wang, H., Sui, N., et al. (2016). Parkin promotes proteasomal degradation of p62: implication of selective vulnerability of neuronal cells in the pathogenesis of Parkinson’s disease. Protein Cell *7*, 114–129.

Tao, Z., Barker, J., Shi, S.D.-H., Gehring, M., and Sun, S. (2010). Steady-State Kinetic and Inhibition Studies of the Mammalian Target of Rapamycin (mTOR) Kinase Domain and mTOR Complexes. Biochemistry *49*, 8488–8498.

Wen, Y., Graybill, W.S., Previs, R.A., Hu, W., Ivan, C., Mangala, L.S., Zand, B., Nick, A.M., Jennings, N.B., Dalton, H.J., et al. (2015). Immunotherapy targeting folate receptor induces cell death associated with autophagy in ovarian cancer. Clin. Cancer Res. Off. J. Am. Assoc. Cancer Res. *21*, 448–459.

Wild, P., Farhan, H., McEwan, D.G., Wagner, S., Rogov, V.V., Brady, N.R., Richter, B., Korac, J., Waidmann, O., Choudhary, C., et al. (2011). Phosphorylation of the Autophagy Receptor Optineurin Restricts Salmonella Growth. Science *333*, 228–233.

Xu, D., Li, X., Shao, F., Lv, G., Lv, H., Lee, J.-H., Qian, X., Wang, Z., Xia, Y., Du, L., et al. (2019). The protein kinase activity of fructokinase A specifies the antioxidant responses of tumor cells by phosphorylating p62. Sci. Adv. *5*, eaav4570.
