# Supplementary material for: Multiplexed proteomics of autophagy-deficient murine macrophages reveals enhanced antimicrobial immunity via the oxidative stress response
Source: eLife. 2021 Jun 4;10:e62320. doi: 10.7554/eLife.62320 (PMC8177894; doi:10.7554/eLife.62320)
Supplement: Supplementary file 3. [file elife-62320-supp3.docx]

| **Supplementary File 3** | |
| --- | --- |
| **Protein name/site** | **Biological significance** |
| **TNFA** | |
| K20 | - Jiang et al.(Jiang et al., 2013) described modification of K20 and K19 by long-chain fatty acids. Removal of fatty chains by SIRT6 promotes TNF-α secretion |
| **TNFR1B/TNFR2** | |
| K300 | - Novel, no reference found |
| **TNFR1A/TNFR1** | |
| No sites reported in our study. |  |
| **BIRC2/cIAP** | |
| S153 | - N/A, not novel |
| S551 | - N/A, not novel |
| **M3K7/TAK1** | |
| S331 | - Novel, no reference found |
| T333 | - N/A, not novel |
| S367 | - N/A, not novel |
| S375 | - N/A, not novel |
| S389 | - A proteomic study identifies phosphorylation at S389 in response to LPS in macrophages(Wu et al., 2019) - This site is also linked to TAK1 activation(Gallardo et al., 2018) |
| S393 | - N/A, not novel |
| S412 | - Phosphorylation S412 is required for TAK1 activation - S412 phosphorylation occurs in response to LPS in RAW264.7 cells(Kobayashi et al., 2005) - S412 phosphorylation in response to TLR activation contributes to innate immune response(Ouyang et al., 2014) - pS412 is linked to constitutive activation of TAK1 in leukemia and allergy(Watson et al., 2020) |
| S412_S427 | - N/A, not novel |
| S428 | - N/A, not novel |
| K447 | - N/A, not novel |
| **TAB2** | |
| S353 | - Novel, no reference found |
| S372 | - Mendoza et al.(Mendoza et al., 2008) described phosphorylation at S372 in response to IL-1 in HEK293 and MEFs |
| T376 | - Novel, no reference found |
| S450 | - Fabrik et.al(Fabrik et al., 2018) reported phosphorylation at S450 in response to infection of bone marrow-derived dendritic cells with Francisella tularensis at late infection stage |
| S524 | - Mendoza et al.(Mendoza et al., 2008) described phosphorylation at S524 site in response to IL-1 in HEK293 and MEFs |
| S527 | - N/A, not novel |
| S580 | - N/A, not novel |
| S582 | - Mendoza et al.(Mendoza et al., 2008) described phosphorylation at S582 site in response to IL-1 in HEK293 and MEFs |
| S584 | - Novel, no reference found |
| K653 | - N/A, not novel |
| **TAB3** | |
| S102 | - N/A, not novel |
| S387_S389 | - N/A, not novel |
| S389 | - N/A, not novel |
| T408 | - Mendoza et al.(Mendoza et al., 2008) described phosphorylation at T408 in response to IL-1 in HEK293 and MEFs - Tao et al.(Tao et al., 2016) reported that phosphorylation at T408 is required for O-GlcNAcylation of S412 |
| S412 | - Tao et al.(Tao et al., 2016) reported S412 O-GlcNAcylation in triple negative breast cancer, which is important for TAK1 activation and influences cell invasion |
| S413 | - N/A, not novel |
| S496 | - N/A, not novel |
| S510 | - Mendoza et al.(Mendoza et al., 2008) describes phosphorylation at S510 in response to IL-1 in HEK293 and MEFs |
| S513 | - N/A, not novel |
| **TRAF1** | |
| S60 | - N/A, not novel |
| S63 | - N/A, not novel |
| S165 | - N/A, not novel |
| K120 | - Novel, no reference found |
| K178 | - This is one of two sites that are ubiquitinated by cIAP2, leading to TRAF1 degradation(Lee et al., 2004) |
| K293 | - Novel, no reference found |
| **TRAF2** | |
| K194 | - Novel, no reference found |
| K313 | - N/A, not novel |
| K331 | - N/A, not novel |
| **NEMO** | |
| S380 | - N/A, not novel |
| K270 | - Huang et al.(Huang et al., 2003) reported sumoylation at K270 and K302 by SUMO-1 in response to DNA damage - Mabb et al.(Mabb et al., 2006) demonstrated K270 and K302 are sumoylated by PIAS in response to genotoxic stress. Interestingly, NEMO sumoylation is also induced by oxidative stress. These events result in NF-kB activation - NEMO sumoylation on k270 is linked to diabetes(Shao et al., 2015) - Liu et al.(Liu et al., 2013) reported that K270 is sumoylated with SUMO-2/3 and desumoylation of this modification by SENP6 is important to dampen activity following TLR3/4 activation |
| K276 | - N/A, not novel |
| K278 | - This residue is ubiquitinated by K63-linked ubiquitin chains in response to NOD2 receptor activation(Abbott et al., 2004). Ubiquitination at K278 is critical for embryonic development(Jun et al., 2013) - Tokunaga et al.(Tokunaga et al., 2009) described ubiquitination of residues K278 and K302 by LUBAC, which is essential for NF-kB activation. Subsequently, Müller-Rischart et al.(Müller-Rischart et al., 2013) reported ubiquitination of these residues by Parkin, which is important response to mitochondrial stress - Hinz et al.(Hinz et al., 2010) reported mono-ubiquitylation at K278 by cIAP1 as a part of genotoxic stress response |
| K302 | - Multiple publications(Huang et al., 2003; Mabb et al., 2006; Shao et al., 2015) reported the significance of K270/K302 sumoylation as described above. Additionally, Niu et al.(Niu et al., 2011) described that K278 and K302 ubiquitination by LUBAC is important step in response to genotoxic stress and that this event is downstream of NEMO nuclear export - See also Müller-Rischart et al.(Müller-Rischart et al., 2013) and Tokunaga et al.(Tokunaga et al., 2009) for NEMO ubiquitination |
| **IKKB** | |
| S670 | - N/A, not novel |
| S672 | - Phosphorylation at S672 was identified in response to LPS(Wu et al., 2019) |
| S675 | - N/A, not novel |
| S679 | - N/A, not novel |
| S689_S692 | - N/A, not novel |
| S693 | - N/A, not novel |
| S697 | - Phosphorylation at S697 was identified in response to LPS(Wu et al., 2019) |
| K498 | - N/A, not novel |
| K555 | - N/A, not novel |
| **IKKE** | |
| S172 | - Kishore et al.(Kishore et al., 2002) and Shimada et al.(Shimada et al., 1999) described that phosphorylation at S172 is required for the catalytic activity |
| S665 | - N/A, not novel |
| S713 | - N/A, not novel |
| K231 | - Sumoylation at this residue is reported in Renner et al.(Renner et al., 2010). Plays an important role in NF-kB activation following genotoxic stress |
| K241 | - N/A, not novel |
| **IKBZ** | |
| T188 | - Novel, no reference found |
| S189 | - N/A, not novel |
| T192 | - N/A, not novel |
| S262 | - N/A, not novel |
| K5 | - Novel, no reference found |
| K120 | - Novel, no reference found |
| K132 | - Novel, no reference found |
| **NFKB1** | |
| S447 | - N/A, not novel |
| S447_S452 | - N/A, not novel |
| S896 | - N/A, not novel |
| S897 | - Fujimoto et al.(Fujimoto et al., 1995) reported phosphorylation at S897 as one of two residues by CDK in vitro |
| T942 | - N/A, not novel |
| K275 | - Novel, no reference found |
| K323 | - N/A, not novel |
| **NFKB2** | |
| S222 | - N/A, not novel |
| T425 | - N/A, not novel |
| S680 | - N/A, not novel |
| T682 | - N/A, not novel |
| S858 | - N/A, not novel |
| S871 | - This residue is a part of sites phosphorylated by IKKα(Qu et al., 2004; Xiao et al., 2004). |
| **REL** | |
| S321 | - Novel, no reference found |
| S488 | - N/A, not novel |
| **RELB** | |
| S552 | - Marienfeld et al.(Marienfeld et al., 2001) and Neumann et al.(Neumann et al., 2011) report RelB S552 phosphorylation by GSK-3β kinase in T cell following TCR stimulation |
| **RNF31/HOIP** | |
| S377 | - N/A, not novel |
| S441 | - Novel, no reference found |
| S445 | - N/A, not novel |
| S973 | - Novel, no reference found |
| K777 | - N/A, not novel |
| K911 | - Novel, no reference found |
| **OTUL** | |
| K34 | - N/A, not novel |
| K64 | - N/A, not novel |
| K66 | - N/A, not novel |
| K116 | - N/A, not novel |
| **TNAP3/A20** | |
| S217 | - Novel, no reference found |
| S220 | - N/A, not novel |
| S381 | - Phosphorylation at this site by IKKβ is an important negative regulation mechanism of the NF-kB pathway(Hutti et al., 2007) - p381, and pS480, pS565, pT625, activate A20 DUB activity against K63-linked chains(Wertz et al., 2015) |
| S498 | - N/A, not novel |
| S534 | - N/A, not novel |
| S535 | - N/A, not novel |
| S558_S563 | - N/A, not novel |
| T567 | - Novel, no reference found |
| S571 | - N/A, not novel |
| S571_S577 | - N/A, not novel |
| S573 | - N/A, not novel |
| S577 | - N/A, not novel |
| S622 | - Novel, no reference found |
| S730 | - Novel, no reference found |
| K31 | - Novel, no reference found |
| K66 | - N/A, not novel |
| K81 | - N/A, not novel |
| K124 | - N/A, not novel |
| K213 | - Novel, no reference found |
| K526 | - N/A, not novel |
| K594 | - N/A, not novel |
| K620 | - N/A, not novel |
| K628 | - N/A, not novel |
| **TNIP1/ABIN1** | |
| S118 | - N/A, not novel |
| S278 | - N/A, not novel |
| S279 | - N/A, not novel |
| S416 | - N/A, not novel |
| S448 | - N/A, not novel |
| S455 | - N/A, not novel |
| S601 | - Novel, no reference found |
| K288 | - Novel, no reference found |
| K314 | - N/A, not novel |
| K317 | - Novel, no reference found |
| K360 | - N/A, not novel |
| K386 | - Novel, no reference found |
| K402 | - N/A, not novel |
| K465 | - N/A, not novel |
| K480 | - N/A, not novel |
| K522 | - N/A, not novel |
| K525 | - N/A, not novel |
| **TNIP2/ABIN2** | |
| S147 | - N/A, not novel |
| S187 | - N/A, not novel |
| T194 | - Novel, no reference found |
| S196 | - Novel, no reference found |
| S414 | - N/A, not novel |
| K358 | - N/A, not novel |
| **MCL1** | |
| S73 | - Several kinases are implicated in phosphorylating multiple Mcl-1 sites. For example, multiple papers(Choudhary et al., 2015; Chu et al., 2016; Domina et al., 2004; Harley et al., 2010; Nikhil and Shah, 2017) showed that phosphorylation at S73 and T144 is CDK-regulated in mitosis, which is important to induce apoptosis if cells cannot resolve mitotic arrest. In addition, Ding et al.(Ding et al., 2008) shows that Mcl1 is also phosphorylated by Erk. - Inuzuka et al.(Inuzuka et al., 2011) and Wertz et al.(Wertz et al., 2011) demonstrated that phosphorylated Mcl1 is degraded and this requires the Fbw7 F-box protein |
| T144 | - Multiple papers(Choudhary et al., 2015; Chu et al., 2016; Harley et al., 2010; Nikhil and Shah, 2017) report this phosphorylation site. - Inoshita et al.(Inoshita et al., 2002) identifies phosphorylation at T144 by JNK in response to oxidative stress |
| K39 | - Ubiquitination at multiple lysines, including K39 and K178, contributes to Mcl1 stability(Ye et al., 2017). |
| K178 | - Ubiquitination at multiple lysines, including K39 and K178, contributes to Mcl1 stability(Ye et al., 2017). |
| K215 | - N/A, not novel |
| K219 | - N/A, not novel |
| **BID** | |
| S78 | - Phosphorylation of BID at S78 by ATM/ATR is important in preserving genome stability(Kamer et al., 2005; Zinkel et al., 2005) |
| K129 | - N/A, not novel |
| K135 | - N/A, not novel |
| K158 | - N/A, not novel |
| **CASP8** | |
| S60 | - Novel, no reference found |
| S188 | - N/A, not novel |
| S198 | - N/A, not novel |
| S213 | - N/A, not novel |
| K33 | - Novel, no reference found |
| K130 | - N/A, not novel |
| K169 | - N/A, not novel |
| K226 | - N/A, not novel |
| K274 | - Novel, no reference found |
| **CFLAR/cFLIP** | |
| K175 | - Novel, no reference found |
| K390 | - Novel, no reference found |
| **RIPK1** | |
| S25 | - N/A, not novel |
| S313 | - N/A, not novel |
| S321 | - Phosphorylation at S321 occurs in response to infection by Yersinia enterocolotica by p38a/MK2 kinase(Dondelinger et al., 2017; Menon et al., 2017). - Phosphorylation at S321 by TAK1 prevents cell death induced by TNF(Geng et al., 2017; Jaco et al., 2017). |
| S415 | - N/A, not novel |
| S595 | - N/A, not novel |
| K20 | - N/A, not novel |
| K307 | - N/A, not novel |
| K429 | - Novel, no reference found |
| K612 | - N/A, not novel |
| K627 | - N/A, not novel |
| **RIPK3** | |
| S173 | - Novel, no reference found |
| S177 | - Novel, no reference found |
| S232 | - Phosphorylation at S232 is required for necrosome activation(Chen et al., 2013; Sun et al., 2012) |
| S254 | - Novel, no reference found |
| S321 | - N/A, not novel |
| T374 | - N/A, not novel |
| S380 | - N/A, not novel |
| T386 | - Novel, no reference found |
| T392 | - Novel, no reference found |
| T398 | - Novel, no reference found |
| T399 | - N/A, not novel |
| T407 | - Novel, no reference found |
| K145 | - Novel, no reference found |
| K230 | - Novel, no reference found |
| K298 | - Novel, no reference found |
| K359 | - Choi et al.(Choi et al., 2018) reported ubiquitylation of human K363 by PELI1 leading to RIP3K degradation |
| **PELI1** | |
| K162 | - N/A, not novel |
| K169 | - N/A, not novel |
| K202 | - K202 is one of five sites reported to be sumoylated *in vitro* and in cell extracts(Kim et al., 2011) |
| **MK08/JNK1** | |
| T183, Y185 | - Dérijard et al.(Dérijard et al., 1994, 1995) identified T183 and Y185 phosphorylation is required for JNK1 activation |
| S377 | - N/A, not novel |
| **JUNB** | |
| S23 | - Bakiri et al.(Bakiri et al., 2000) reported S23 phosphorylation in mitosis |
| T104 | - Phosphorylation of T104 regulates IL-4 expression in Th2 cells(Li et al., 1999) |
| S117 | - N/A, not novel |
| S234 | - N/A, not novel |
| S248, T252, S256 | - Phosphorylation of these residues by GSK3 leads to degradation of JunB in SCF-dependent manner in G2 phase of the cell cycle(Lee et al., 2013; Pérez-Benavente et al., 2013) |
| K36 | - N/A, not novel |
| K237 | - N/A, not novel |
| K322 | - N/A, not novel |
| **DDX58/RIG-I** | |
| K256 | - Novel, no reference found |
| K257 | - N/A, not novel |
| K259 | - N/A, not novel |
| K456 | - N/A, not novel |
| K645 | - N/A, not novel |
| **MAVS** | |
| S179 | - N/A, not novel |
| S186 | - N/A, not novel |
| S220 | - N/A, not novel |
| T222 | - N/A, not novel |
| Y332 | - Novel, no reference found |
| S384 | - N/A, not novel |
| **NOD2** | |
| No sites reported in our study. |  |
| **CGAS** | |
| S34 | - N/A, not novel |
| K55 | - Novel, no reference found |
| K486 | - N/A, not novel |
| **STING** | |
| No sites reported in our study. | ­ |
| **XIAP** | |
| S346 | - N/A, not novel |
| S429 | - Phosphorylation at S429 reported as a response to viral infection by Nakhaei et al.(Nakhaei et al., 2012). |
| K31 | - N/A, not novel |
| K208 | - N/A, not novel |
| K296 | - N/A, not novel |
| K357 | - N/A, not novel |
| **RIPK2** | |
| S183 | - Novel, no reference found |
| S364 | - N/A, not novel |
| S373 | - N/A, not novel |
| S381 | - Novel, no reference found |
| S414 | - N/A, not novel |
| K369 | - Novel, no reference found |
| **TRAF3** | |
| K334 | - N/A, not novel |
| K368 | - N/A, not novel |
| K512 | - N/A, not novel |
| **TRAF6** | |
| No sites reported in our study. |  |
| **MYD88** | |
| S136 | - Novel, no reference found |
| K127 | - N/A, not novel |
| K250 | - N/A, not novel |
| K256 | - N/A, not novel |
| K262 | - N/A, not novel |
| K291 | - N/A, not novel |
| **IRAK1** | |
| S185 | - N/A, not novel |
| K371 | - N/A, not novel |
| K397 | - N/A, not novel |
| **IRAK2** | |
| S136 | - N/A, not novel |
| T140 | - N/A, not novel |
| S168 | - N/A, not novel |
| S175 | - Novel, no reference found |
| T587 | - Novel, no reference found |
| S616 | - Novel, no reference found |
| **IRAK3** | |
| K60 | - Novel, no reference found |
| K163 | - Novel, no reference found |
| K392 | - N/A, not novel |
| **IRAK4** | |
| T133 | - Novel, no reference found |
| S134 | - Novel, no reference found |
| S152 | - N/A, not novel |
| S175_S186 | - Novel, no reference found |
| **TBK1** | |
| S509 | - Novel, no reference found |
| S511 | - N/A, not novel |
| S716 | - N/A, not novel |
| **IRF3** | |
| Y107 | - N/A, not novel |
| S123 | - MAPK mediates phosphorylation at S123 during anti-viral response(Schmid et al., 2014; Wang et al., 2014) |
| T126 | - Novel, no reference found |
| S130 | - Novel, no reference found |
| S131 | - N/A, not novel |
| S135 | - S135 is phosphorylated by DNA-PK during viral response(Karpova et al., 2002) |
| S378, S379, S388, S394 | - Phosphorylation of this patch by multiple kinases including IKKε and TBK1 is critical for triggering virus-induced activation of IRF3-driven gene expression(Mori et al., 2004; Panne et al., 2007; Yoneyama et al., 1998) |
| K70 | - Contributes to IRF3 regulation by sumoylation and ubiquitination(Ran et al., 2011) |
| **IRF7** | |
| S227 | - Novel, no reference found |
| T277 | - Novel, no reference found |
| **TLR2** | |
| No sites reported in our study. |  |
| **TLR4** | |
| K692 | - Novel, no reference found |
| **IFIT1** | |
| S272 | - Novel, no reference found |
| S296 | - Novel, no reference found |
| K89 | - Novel, no reference found |
| K117 | - Novel, no reference found |
| K123 | - Novel, no reference found |
| K406 | - Novel, no reference found |
| K451 | - Novel, no reference found |
| **IFIT2** | |
| K41 | - Novel, no reference found |
| K61 | - Novel, no reference found |
| K126 | - N/A, not novel |
| K158 | - Novel, no reference found |
| K163 | - N/A, not novel |
| K239 | - N/A, not novel |
| K291 | - Novel, no reference found |
| K357 | - N/A, not novel |
| **IFIT3** | |
| S327 | - Novel, no reference found |
| S333 | - Novel, no reference found |
| K189 | - N/A, not novel |
| K236 | - N/A, not novel |
| K246 | - Novel, no reference found |
| K252 | - Novel, no reference found |
| K266 | - Novel, no reference found |
| K396 | - Novel, no reference found |
| **ISG15** | |
| K30 | - Novel, no reference found |
| K35 | - N/A, not novel |
| K141 | - N/A, not novel |
| K148 | - N/A, not novel |
| **OASL1** | |
| No sites reported in our study. |  |
| **GBP1** | |
| K372 | - N/A, not novel |
| **GBP2** | |
| K389 | - N/A, not novel |
| K396 | - N/A, not novel |
| K444 | - N/A, not novel |
| K551 | - N/A, not novel |
| **GBP5** | |
| K279 | - Novel, no reference found |

**References**

Abbott, D.W., Wilkins, A., Asara, J.M., and Cantley, L.C. (2004). The Crohn's disease protein, NOD2, requires RIP2 in order to induce ubiquitinylation of a novel site on NEMO. Curr Biol *14*, 2217–2227.

Bakiri, L., Lallemand, D., Bossy-Wetzel, E., and Yaniv, M. (2000). Cell cycle-dependent variations in c-Jun and JunB phosphorylation: a role in the control of cyclin D1 expression. Embo J *19*, 2056–2068.

Chen, W., Zhou, Z., Li, L., Zhong, C.-Q., Zheng, X., Wu, X., Zhang, Y., Ma, H., Huang, D., Li, W., et al. (2013). Diverse sequence determinants control human and mouse receptor interacting protein 3 (RIP3) and mixed lineage kinase domain-like (MLKL) interaction in necroptotic signaling. Journal of Biological Chemistry *288*, 16247–16261.

Choi, S.-W., Park, H.-H., Kim, S., Chung, J.M., Noh, H.-J., Kim, S.K., Song, H.K., Lee, C.-W., Morgan, M.J., Kang, H.C., et al. (2018). PELI1 Selectively Targets Kinase-Active RIP3 for Ubiquitylation-Dependent Proteasomal Degradation. Mol Cell *70*, 920–935.e927.

Choudhary, G.S., Tat, T.T., Misra, S., Hill, B.T., Smith, M.R., Almasan, A., and Mazumder, S. (2015). Cyclin E/Cdk2-dependent phosphorylation of Mcl-1 determines its stability and cellular sensitivity to BH3 mimetics. Oncotarget *6*, 16912–16925.

Chu, R., Alford, S.E., Hart, K., Kothari, A., Mackintosh, S.G., Kovak, M.R., and Chambers, T.C. (2016). Mitotic arrest-induced phosphorylation of Mcl-1 revisited using two-dimensional gel electrophoresis and phosphoproteomics: nine phosphorylation sites identified. Oncotarget *7*, 78958–78970.

Dérijard, B., Hibi, M., Wu, I.H., Barrett, T., Su, B., Deng, T., Karin, M., and Davis, R.J. (1994). JNK1: a protein kinase stimulated by UV light and Ha-Ras that binds and phosphorylates the c-Jun activation domain. Cell *76*, 1025–1037.

Dérijard, B., Raingeaud, J., Barrett, T., Wu, I.H., Han, J., Ulevitch, R.J., and Davis, R.J. (1995). Independent human MAP-kinase signal transduction pathways defined by MEK and MKK isoforms. Science (New York, NY) *267*, 682–685.

Ding, Q., Huo, L., Yang, J.-Y., Xia, W., Wei, Y., Liao, Y., Chang, C.-J., Yang, Y., Lai, C.-C., Lee, D.-F., et al. (2008). Down-regulation of myeloid cell leukemia-1 through inhibiting Erk/Pin 1 pathway by sorafenib facilitates chemosensitization in breast cancer. Cancer Research *68*, 6109–6117.

Domina, A.M., Vrana, J.A., Gregory, M.A., Hann, S.R., and Craig, R.W. (2004). MCL1 is phosphorylated in the PEST region and stabilized upon ERK activation in viable cells, and at additional sites with cytotoxic okadaic acid or taxol. Oncogene *23*, 5301–5315.

Dondelinger, Y., Delanghe, T., Rojas-Rivera, D., Priem, D., Delvaeye, T., Bruggeman, I., Van Herreweghe, F., Vandenabeele, P., and Bertrand, M.J.M. (2017). MK2 phosphorylation of RIPK1 regulates TNF-mediated cell death. Nat Cell Biol *19*, 1237–1247.

Fabrik, I., Link, M., Putzova, D., Plzakova, L., Lubovska, Z., Philimonenko, V., Pavkova, I., Rehulka, P., Krocova, Z., Hozak, P., et al. (2018). The Early Dendritic Cell Signaling Induced by Virulent Francisella tularensis Strain Occurs in Phases and Involves the Activation of Extracellular Signal-Regulated Kinases (ERKs) and p38 In the Later Stage. Mol Cell Proteomics *17*, 81–94.

Fujimoto, K., Yasuda, H., Sato, Y., and Yamamoto, K. (1995). A role for phosphorylation in the proteolytic processing of the human NF-kappa B1 precursor. Gene *165*, 183–189.

Gallardo, F., Bertran, J., López-Arribillaga, E., González, J., Menéndez, S., Sánchez, I., Colomo, L., Iglesias, M., Garrido, M., Santamaría-Babí, L.F., et al. (2018). Novel phosphorylated TAK1 species with functional impact on NF-κB and β-catenin signaling in human Cutaneous T-cell lymphoma. Leukemia *32*, 2211–2223.

Geng, J., Ito, Y., Shi, L., Amin, P., Chu, J., Ouchida, A.T., Mookhtiar, A.K., Zhao, H., Xu, D., Shan, B., et al. (2017). Regulation of RIPK1 activation by TAK1-mediated phosphorylation dictates apoptosis and necroptosis. Nat Commun *8*, 359–12.

Harley, M.E., Allan, L.A., Sanderson, H.S., and Clarke, P.R. (2010). Phosphorylation of Mcl-1 by CDK1-cyclin B1 initiates its Cdc20-dependent destruction during mitotic arrest. Embo J *29*, 2407–2420.

Hinz, M., Stilmann, M., Arslan, S.Ç., Khanna, K.K., Dittmar, G., and Scheidereit, C. (2010). A cytoplasmic ATM-TRAF6-cIAP1 module links nuclear DNA damage signaling to ubiquitin-mediated NF-κB activation. Mol Cell *40*, 63–74.

Huang, T.T., Wuerzberger-Davis, S.M., Wu, Z.-H., and Miyamoto, S. (2003). Sequential modification of NEMO/IKKgamma by SUMO-1 and ubiquitin mediates NF-kappaB activation by genotoxic stress. Cell *115*, 565–576.

Hutti, J.E., Turk, B.E., Asara, J.M., Ma, A., Cantley, L.C., and Abbott, D.W. (2007). IkappaB kinase beta phosphorylates the K63 deubiquitinase A20 to cause feedback inhibition of the NF-kappaB pathway. Mol Cell Biol *27*, 7451–7461.

Inoshita, S., Takeda, K., Hatai, T., Terada, Y., Sano, M., Hata, J., Umezawa, A., and Ichijo, H. (2002). Phosphorylation and inactivation of myeloid cell leukemia 1 by JNK in response to oxidative stress. J Biol Chem *277*, 43730–43734.

Inuzuka, H., Shaik, S., Onoyama, I., Gao, D., Tseng, A., Maser, R.S., Zhai, B., Wan, L., Gutierrez, A., Lau, A.W., et al. (2011). SCF(FBW7) regulates cellular apoptosis by targeting MCL1 for ubiquitylation and destruction. Nature *471*, 104–109.

Jaco, I., Annibaldi, A., Lalaoui, N., Wilson, R., Tenev, T., Laurien, L., Kim, C., Jamal, K., Wicky John, S., Liccardi, G., et al. (2017). MK2 Phosphorylates RIPK1 to Prevent TNF-Induced Cell Death. Mol Cell *66*, 698–710.e5.

Jiang, H., Khan, S., Wang, Y., Charron, G., He, B., Sebastian, C., Du, J., Kim, R., Ge, E., Mostoslavsky, R., et al. (2013). SIRT6 regulates TNF-α secretion through hydrolysis of long-chain fatty acyl lysine. Nature *496*, 110–113.

Jun, J.C., Kertesy, S., Jones, M.B., Marinis, J.M., Cobb, B.A., Tigno-Aranjuez, J.T., and Abbott, D.W. (2013). Innate immune-directed NF-κB signaling requires site-specific NEMO ubiquitination. CellReports *4*, 352–361.

Kamer, I., Sarig, R., Zaltsman, Y., Niv, H., Oberkovitz, G., Regev, L., Haimovich, G., Lerenthal, Y., Marcellus, R.C., and Gross, A. (2005). Proapoptotic BID is an ATM effector in the DNA-damage response. Cell *122*, 593–603.

Karpova, A.Y., Trost, M., Murray, J.M., Cantley, L.C., and Howley, P.M. (2002). Interferon regulatory factor-3 is an in vivo target of DNA-PK. Proc Natl Acad Sci USA *99*, 2818–2823.

Kim, J.H., Sung, K.S., Jung, S.M., Lee, Y.S., Kwon, J.Y., Choi, C.Y., and Park, S.H. (2011). Pellino-1, an adaptor protein of interleukin-1 receptor/toll-like receptor signaling, is sumoylated by Ubc9. Mol. Cells *31*, 85–89.

Kishore, N., Huynh, Q.K., Mathialagan, S., Hall, T., Rouw, S., Creely, D., Lange, G., Caroll, J., Reitz, B., Donnelly, A., et al. (2002). IKK-i and TBK-1 are enzymatically distinct from the homologous enzyme IKK-2: comparative analysis of recombinant human IKK-i, TBK-1, and IKK-2. J Biol Chem *277*, 13840–13847.

Kobayashi, Y., Mizoguchi, T., Take, I., Kurihara, S., Udagawa, N., and Takahashi, N. (2005). Prostaglandin E2 enhances osteoclastic differentiation of precursor cells through protein kinase A-dependent phosphorylation of TAK1. J Biol Chem *280*, 11395–11403.

Lee, J.K.H., Pearson, J.D., Maser, B.E., and Ingham, R.J. (2013). Cleavage of the JunB transcription factor by caspases generates a carboxyl-terminal fragment that inhibits activator protein-1 transcriptional activity. Journal of Biological Chemistry *288*, 21482–21495.

Lee, J.-S., Hong, U.-S., Lee, T.H., Yoon, S.K., and Yoon, J.-B. (2004). Mass spectrometric analysis of tumor necrosis factor receptor-associated factor 1 ubiquitination mediated by cellular inhibitor of apoptosis 2. Proteomics *4*, 3376–3382.

Li, B., Tournier, C., Davis, R.J., and Flavell, R.A. (1999). Regulation of IL-4 expression by the transcription factor JunB during T helper cell differentiation. Embo J *18*, 420–432.

Liu, X., Chen, W., Wang, Q., Li, L., and Wang, C. (2013). Negative regulation of TLR inflammatory signaling by the SUMO-deconjugating enzyme SENP6. PLoS Pathog *9*, e1003480.

Mabb, A.M., Wuerzberger-Davis, S.M., and Miyamoto, S. (2006a). PIASy mediates NEMO sumoylation and NF-kappaB activation in response to genotoxic stress. Nat Cell Biol *8*, 986–993.

Mabb, A.M., Wuerzberger-Davis, S.M., and Miyamoto, S. (2006b). PIASy mediates NEMO sumoylation and NF-κB activation in response to genotoxic stress. Nat Cell Biol *8*, 986–993.

Marienfeld, R., Berberich-Siebelt, F., Berberich, I., Denk, A., Serfling, E., and Neumann, M. (2001). Signal-specific and phosphorylation-dependent RelB degradation: a potential mechanism of NF-kappaB control. Oncogene *20*, 8142–8147.

Mendoza, H., Campbell, D.G., Burness, K., Hastie, J., Ronkina, N., Shim, J.-H., Arthur, J.S.C., Davis, R.J., Gaestel, M., Johnson, G.L., et al. (2008). Roles for TAB1 in regulating the IL-1-dependent phosphorylation of the TAB3 regulatory subunit and activity of the TAK1 complex. Biochem J *409*, 711–722.

Menon, M.B., Gropengießer, J., Fischer, J., Novikova, L., Deuretzbacher, A., Lafera, J., Schimmeck, H., Czymmeck, N., Ronkina, N., Kotlyarov, A., et al. (2017). p38MAPK/MK2-dependent phosphorylation controls cytotoxic RIPK1 signalling in inflammation and infection. Nat Cell Biol *19*, 1248–1259.

Mori, M., Yoneyama, M., Ito, T., Takahashi, K., Inagaki, F., and Fujita, T. (2004). Identification of Ser-386 of interferon regulatory factor 3 as critical target for inducible phosphorylation that determines activation. J Biol Chem *279*, 9698–9702.

Müller-Rischart, A.K., Pilsl, A., Beaudette, P., Patra, M., Hadian, K., Funke, M., Peis, R., Deinlein, A., Schweimer, C., Kuhn, P.-H., et al. (2013). The E3 ligase parkin maintains mitochondrial integrity by increasing linear ubiquitination of NEMO. Mol Cell *49*, 908–921.

Nakhaei, P., Sun, Q., Solis, M., Mesplede, T., Bonneil, E., Paz, S., Lin, R., and Hiscott, J. (2012). IκB kinase ε-dependent phosphorylation and degradation of X-linked inhibitor of apoptosis sensitizes cells to virus-induced apoptosis. J Virol *86*, 726–737.

Neumann, M., Klar, S., Wilisch-Neumann, A., Hollenbach, E., Kavuri, S., Leverkus, M., Kandolf, R., Brunner-Weinzierl, M.C., and Klingel, K. (2011). Glycogen synthase kinase-3β is a crucial mediator of signal-induced RelB degradation. Oncogene *30*, 2485–2492.

Nikhil, K., and Shah, K. (2017). The Cdk5-Mcl-1 axis promotes mitochondrial dysfunction and neurodegeneration in a model of Alzheimer's disease. J Cell Sci *130*, 3023–3039.

Niu, J., Shi, Y., Iwai, K., and Wu, Z.-H. (2011). LUBAC regulates NF-κB activation upon genotoxic stress by promoting linear ubiquitination of NEMO. Embo J *30*, 3741–3753.

Ouyang, C., Nie, L., Gu, M., Wu, A., Han, X., Wang, X., Shao, J., and Xia, Z. (2014). Transforming growth factor (TGF)-β-activated kinase 1 (TAK1) activation requires phosphorylation of serine 412 by protein kinase A catalytic subunit α (PKACα) and X-linked protein kinase (PRKX). Journal of Biological Chemistry *289*, 24226–24237.

Panne, D., McWhirter, S.M., Maniatis, T., and Harrison, S.C. (2007). Interferon regulatory factor 3 is regulated by a dual phosphorylation-dependent switch. J Biol Chem *282*, 22816–22822.

Pérez-Benavente, B., García, J.L., Rodríguez, M.S., Pineda-Lucena, A., Piechaczyk, M., Font de Mora, J., and Farràs, R. (2013). GSK3-SCF(FBXW7) targets JunB for degradation in G2 to preserve chromatid cohesion before anaphase. Oncogene *32*, 2189–2199.

Qu, Z., Qing, G., Rabson, A., and Xiao, G. (2004). Tax deregulation of NF-kappaB2 p100 processing involves both beta-TrCP-dependent and -independent mechanisms. J Biol Chem *279*, 44563–44572.

Ran, Y., Liu, T.-T., Zhou, Q., Li, S., Mao, A.-P., Li, Y., Liu, L.-J., Cheng, J.-K., and Shu, H.-B. (2011). SENP2 negatively regulates cellular antiviral response by deSUMOylating IRF3 and conditioning it for ubiquitination and degradation. J Mol Cell Biol *3*, 283–292.

Renner, F., Moreno, R., and Schmitz, M.L. (2010). SUMOylation-dependent localization of IKKepsilon in PML nuclear bodies is essential for protection against DNA-damage-triggered cell death. Mol Cell *37*, 503–515.

Schmid, S., Sachs, D., and tenOever, B.R. (2014). Mitogen-activated protein kinase-mediated licensing of interferon regulatory factor 3/7 reinforces the cell response to virus. Journal of Biological Chemistry *289*, 299–311.

Shao, L., Zhou, H.J., Zhang, H., Qin, L., Hwa, J., Yun, Z., Ji, W., and Min, W. (2015). SENP1-mediated NEMO deSUMOylation in adipocytes limits inflammatory responses and type-1 diabetes progression. Nat Commun *6*, 8917–8920.

Shimada, T., Kawai, T., Takeda, K., Matsumoto, M., Inoue, J., Tatsumi, Y., Kanamaru, A., and Akira, S. (1999). IKK-i, a novel lipopolysaccharide-inducible kinase that is related to IkappaB kinases. Int. Immunol. *11*, 1357–1362.

Sun, L., Wang, H., Wang, Z., He, S., Chen, S., Liao, D., Wang, L., Yan, J., Liu, W., Lei, X., et al. (2012). Mixed lineage kinase domain-like protein mediates necrosis signaling downstream of RIP3 kinase. Cell *148*, 213–227.

Tao, T., He, Z., Shao, Z., and Lu, H. (2016). TAB3 O-GlcNAcylation promotes metastasis of triple negative breast cancer. Oncotarget *7*, 22807–22818.

Tokunaga, F., Sakata, S.-I., Saeki, Y., Satomi, Y., Kirisako, T., Kamei, K., Nakagawa, T., Kato, M., Murata, S., Yamaoka, S., et al. (2009). Involvement of linear polyubiquitylation of NEMO in NF-kappaB activation. Nature Cell Biology *11*, 123–132.

Wang, J.-T., Chang, L.-S., Chen, C.-J., Doong, S.-L., Chang, C.-W., and Chen, M.-R. (2014). Glycogen synthase kinase 3 negatively regulates IFN regulatory factor 3 transactivation through phosphorylation at its linker region. Innate Immunity *20*, 78–87.

Watson, C.J.F., Maguire, A.R.R., Rouillard, M.M., Crozier, R.W.E., Yousef, M., Bruton, K.M., Fajardo, V.A., and MacNeil, A.J. (2020). TAK1 signaling activity links the mast cell cytokine response and degranulation in allergic inflammation. J. Leukoc. Biol. *107*, 649–661.

Wertz, I.E., Kusam, S., Lam, C., Okamoto, T., Sandoval, W., Anderson, D.J., Helgason, E., Ernst, J.A., Eby, M., Liu, J., et al. (2011). Sensitivity to antitubulin chemotherapeutics is regulated by MCL1 and FBW7. Nature *471*, 110–114.

Wertz, I.E., Newton, K., Seshasayee, D., Kusam, S., Lam, C., Zhang, J., Popovych, N., Helgason, E., Schoeffler, A., Jeet, S., et al. (2015). Phosphorylation and linear ubiquitin direct A20 inhibition of inflammation. Nature *528*, 370–375.

Wu, X., Yang, D., Zhao, F., Yang, Z.-H., Wang, D., Qiao, M., Fang, Y., Li, W., Wu, R., He, P., et al. (2019). Quantification of Dynamic Protein Interactions and Phosphorylation in LPS Signaling Pathway by SWATH-MS. Mol Cell Proteomics *18*, 1054–1069.

Xiao, G., Fong, A., and Sun, S.-C. (2004). Induction of p100 processing by NF-kappaB-inducing kinase involves docking IkappaB kinase alpha (IKKalpha) to p100 and IKKalpha-mediated phosphorylation. J Biol Chem *279*, 30099–30105.

Ye, M., Zhang, Y., Zhang, X., Zhang, J., Jing, P., Cao, L., Li, N., Li, X., Yao, L., and Zhang, J. (2017). Targeting FBW7 as a Strategy to Overcome Resistance to Targeted Therapy in Non-Small Cell Lung Cancer. Cancer Research *77*, 3527–3539.

Yoneyama, M., Suhara, W., Fukuhara, Y., Fukuda, M., Nishida, E., and Fujita, T. (1998). Direct triggering of the type I interferon system by virus infection: activation of a transcription factor complex containing IRF-3 and CBP/p300. Embo J *17*, 1087–1095.

Zinkel, S.S., Hurov, K.E., Ong, C., Abtahi, F.M., Gross, A., and Korsmeyer, S.J. (2005). A role for proapoptotic BID in the DNA-damage response. Cell *122*, 579–591.
