# Supplementary material for: Multiplexed proteomics of autophagy-deficient murine macrophages reveals enhanced antimicrobial immunity via the oxidative stress response
Source: eLife. 2021 Jun 4;10:e62320. doi: 10.7554/eLife.62320 (PMC8177894; doi:10.7554/eLife.62320)
Supplement: Transparent reporting form [file elife-62320-transrepform.docx]

***eLife’s* transparent reporting form**

We encourage authors to provide detailed information *within their submission* to facilitate the interpretation and replication of experiments. Authors can upload supporting documentation to indicate the use of appropriate reporting guidelines for health-related research (see [EQUATOR Network](http://www.equator-network.org/%20)), life science research (see the [BioSharing Information Resource](https://biosharing.org/)), or the [ARRIVE guidelines](http://www.plosbiology.org/article/info:doi/10.1371/journal.pbio.1000412) for reporting work involving animal research. Where applicable, authors should refer to any relevant reporting standards documents in this form.

If you have any questions, please consult our Journal Policies and/or contact us: [editorial@elifesciences.org](mailto:editorial@elifesciences.org).

**Sample-size estimation**

- You should state whether an appropriate sample size was computed when the study was being designed
- You should state the statistical method of sample size computation and any required assumptions
- If no explicit power analysis was used, you should describe how you decided what sample (replicate) size (number) to use

Please outline where this information can be found within the submission (e.g., sections or figure legends), or explain why this information doesn’t apply to your submission:

Sample sizes are directly depicted in each plot (dots or histogram), and numbers are provided within each figure legend. No statistical method was used for sample size computation or any assumptions since all cell- and animal-based studies were performed using genetically identical groups for comparison.

**Replicates**

- You should report how often each experiment was performed
- You should include a definition of biological versus technical replication
- The data obtained should be provided and sufficient information should be provided to indicate the number of independent biological and/or technical replicates
- If you encountered any outliers, you should describe how these were handled
- Criteria for exclusion/inclusion of data should be clearly stated
- High-throughput sequence data should be uploaded before submission, with a private link for reviewers provided (these are available from both GEO and ArrayExpress)

Please outline where this information can be found within the submission (e.g., sections or figure legends), or explain why this information doesn’t apply to your submission:

Replicate information is provided within each figure legend. Biological (separate mice, independent experiments) versus technical (number per experiment) replicates are detailed in each figure. All mass spectrometry datasets were generated with a biological quadruplicate (n=4), performed in two independent experiments (termed ‘Plexes’) each executed with biological duplicates. This detail is provided in the Materials and Methods. Outlier exclusion criteria are detailed in the figure legend for relevant datasets. All mass spectrometry data is uploaded to UCSF MassIVE; URL and password is provided within the Data availability statement.

**Statistical reporting**

- Statistical analysis methods should be described and justified
- Raw data should be presented in figures whenever informative to do so (typically when N per group is less than 10)
- For each experiment, you should identify the statistical tests used, exact values of N, definitions of center, methods of multiple test correction, and dispersion and precision measures (e.g., mean, median, SD, SEM, confidence intervals; and, for the major substantive results, a measure of effect size (e.g., Pearson's r, Cohen's d)
- Report exact p-values wherever possible alongside the summary statistics and 95% confidence intervals. These should be reported for all key questions and not only when the p-value is less than 0.05.

Please outline where this information can be found within the submission (e.g., sections or figure legends), or explain why this information doesn’t apply to your submission:

Statistical analysis methods used in each experiment are stated in the respective figure legend. Statistical methods for mass spectrometry studies are detailed in the Materials and Methods section. All histograms and dot plots depict raw data. Supplementary tables 6-9 provide MSStatsTMT quantification and statistical testing results for the mass spectrometry datasets.

(For large datasets, or papers with a very large number of statistical tests, you may upload a single table file with tests, Ns, etc., with reference to sections in the manuscript.)

**Group allocation**

- Indicate how samples were allocated into experimental groups (in the case of clinical studies, please specify allocation to treatment method); if randomization was used, please also state if restricted randomization was applied
- Indicate if masking was used during group allocation, data collection and/or data analysis

Please outline where this information can be found within the submission (e.g., sections or figure legends), or explain why this information doesn’t apply to your submission:

Samples were allocated into experimental groups based on genotype and treatment (infection) conditions. No randomization was applied. No masking was used during group allocation, data collection or analysis.

**Additional data files (“source data”)**

- We encourage you to upload relevant additional data files, such as numerical data that are represented as a graph in a figure, or as a summary table
- Where provided, these should be in the most useful format, and they can be uploaded as “Source data” files linked to a main figure or table
- Include model definition files including the full list of parameters used
- Include code used for data analysis (e.g., R, MatLab)
- Avoid stating that data files are “available upon request”

Please indicate the figures or tables for which source data files have been provided:

All mass spectrometry quantification reports are uploaded as Supplementary files 5, 7, and 9. Mass spectrometry data is uploaded to UCSD MassIVE and URL/password provided within the Data availability statement. All code is made available via appropriate URLs in the Software availability statement. Readers can access and visualize mass spectrometry data in a new interactive resource we have made publicly available (URL listed in the Discussion: <https://info.perkinelmer.com/analytics-resource-center>).
